# A digital microfluidic system for loop-mediated isothermal amplification and sequence specific pathogen detection

**DOI:** 10.1038/s41598-017-14698-x

**Published:** 2017-11-06

**Authors:** Liang Wan, Tianlan Chen, Jie Gao, Cheng Dong, Ada Hang-Heng Wong, Yanwei Jia, Pui-In Mak, Chu-Xia Deng, Rui P. Martins

**Affiliations:** 1 State-Key Laboratory of Analog and Mixed-Signal VLSI, University of Macau, Macao, China; 2 Department of ECE, Faculty of Science and Technology, University of Macau, Macao, China; 3 Cancer Centre, Faculty of Health Sciences, University of Macau, Macao, China; 4 Instituto Superior Técnico, Universidade de Lisboa, Lisboa, Portugal

## Abstract

A digital microfluidic (DMF) system has been developed for loop-mediated isothermal amplification (LAMP)-based pathogen nucleic acid detection using specific low melting temperature (T_m_) Molecular Beacon DNA probes. A positive-temperature-coefficient heater with a temperature sensor for real-time thermal regulation was integrated into the control unit, which generated actuation signals for droplet manipulation. To enhance the specificity of the LAMP reaction, low-T_m_ Molecular Beacon probes were designed within the single-stranded loop structures on the LAMP reaction products. In the experiments, only 1 μL of LAMP reaction samples containing purified *Trypanosoma brucei* DNA were required, which represented over a 10x reduction of reagent consumption when comparing with the conventional off-chip LAMP. On-chip LAMP for unknown sample detection could be accomplished in 40 min with a detection limit of 10 copies/reaction. Also, we accomplished an on-chip melting curve analysis of the Molecular Beacon probe from 30 to 75 °C within 5 min, which was 3x faster than using a commercial qPCR machine. Discrimination of non-specific amplification and lower risk of aerosol contamination for on-chip LAMP also highlight the potential utilization of this system in clinical applications. The entire platform is open for further integration with sample preparation and fluorescence detection towards a total-micro-analysis system.

## Introduction

Digital microfluidics (DMF) is an emerging technology in the microfluidic field to manipulate individual microliter- to nanoliter-sized droplets on an array of electrodes by electro-wetting force^1^. The special features of this technology including miniaturization of biochemical reactions to allow low reagent input, fast heat transfer, highly reconfigurable droplet control, and small footprints have made it a promising candidate for point-of-care testing (POCT). Numerous DMF studies have been devoted to biochemical analyte analysis^2,3^, immunoassays^4,5^, and molecular diagnostics^6,7^.

Loop-mediated isothermal amplification (LAMP) is a favorite molecular diagnostic method in the field of POCT^8–11^ because it works at a constant temperature with high efficiency, thereby significantly simplifying reaction equipment requirements. Invented and furthered developed since 2000^12^, LAMP has salient advantages over other nucleic acid amplification methods in terms of low cost, rapidity, robustness, high sensitivity and specificity, simple product detection by the naked eye, and isothermal reaction conditions^13^. Driven by the need for POCT in low resource settings, various product detection techniques have been developed to take advantage of the high levels of DNA amplicons produced by LAMP, including precipitation with magnesium pyrophosphate, staining with fluorescence intercalating dye, Mg^2+^ indicator calcein and hydroxy naphthol blue^14–17^. But, all of these detection methods are based on the mass of DNA produced by the LAMP reaction, regardless of whether the product is sequence specific or non-specific.

False-positives result from non-specific amplification, primer-dimer formation, or contamination. Non-specific amplification is a risk for any DNA amplification methodology. Although LAMP uses 4–6 primers that recognize 6–8 genomic loci to enhance the detection specificity^12,18^, non-specific amplification occurs frequently^19,20^ owing to the high concentration of primers and Mg^2+^. Efforts have been made to discriminate non-specific amplification using nucleic acid probes that targeted specific single-stranded sequences in the loop structures of the LAMP products, such as biotinylated probes used in southern blotting^21^, oligo DNA probes with cationic polymers enabling naked-eyed visualization^22^, FITC-labeled probes utilized in lateral flow dipstick^23^, alternative binding quenching probes^24^, and fluorescence energy transfer-based probes^25^. But these additional technologies either require post-amplification manipulation, which may lead to cross contamination, or involve complicated probe designs and assay settings.

Molecular Beacon DNA probes, in contrast, are much easier to design and more easily detected because their background fluorescence is low. Each Molecular Beacon has a stem-loop structure bringing both ends in close proximity enabling contact quenching^26^. Upon hybridization of the loop of the probe to its target, the fluorophore and the quencher are separated permitting the fluorophore to fluoresce. Molecular Beacon probes have been widely used to detect single-stranded PCR products^27,28^ with single nucleotide variation based on melting curve analysis (MCA)^7^. Thus, the single-stranded loop structures in LAMP products make them ideal targets for Molecular Beacon assays. Recently, Liu *et al*. investigated the use of a Molecular Beacon probe to monitor real-time LAMP reactions^29^. However, real-time monitoring of probe signals required probe T_m_ (melting temperature) to be near or higher than the reaction temperature so that probes could bind with the targets and emit fluorescence during the reaction. This protocol, however, lowers reaction efficiency since the Molecular Beacon probes has to be displaced during DNA amplification.

**Figure 1:**
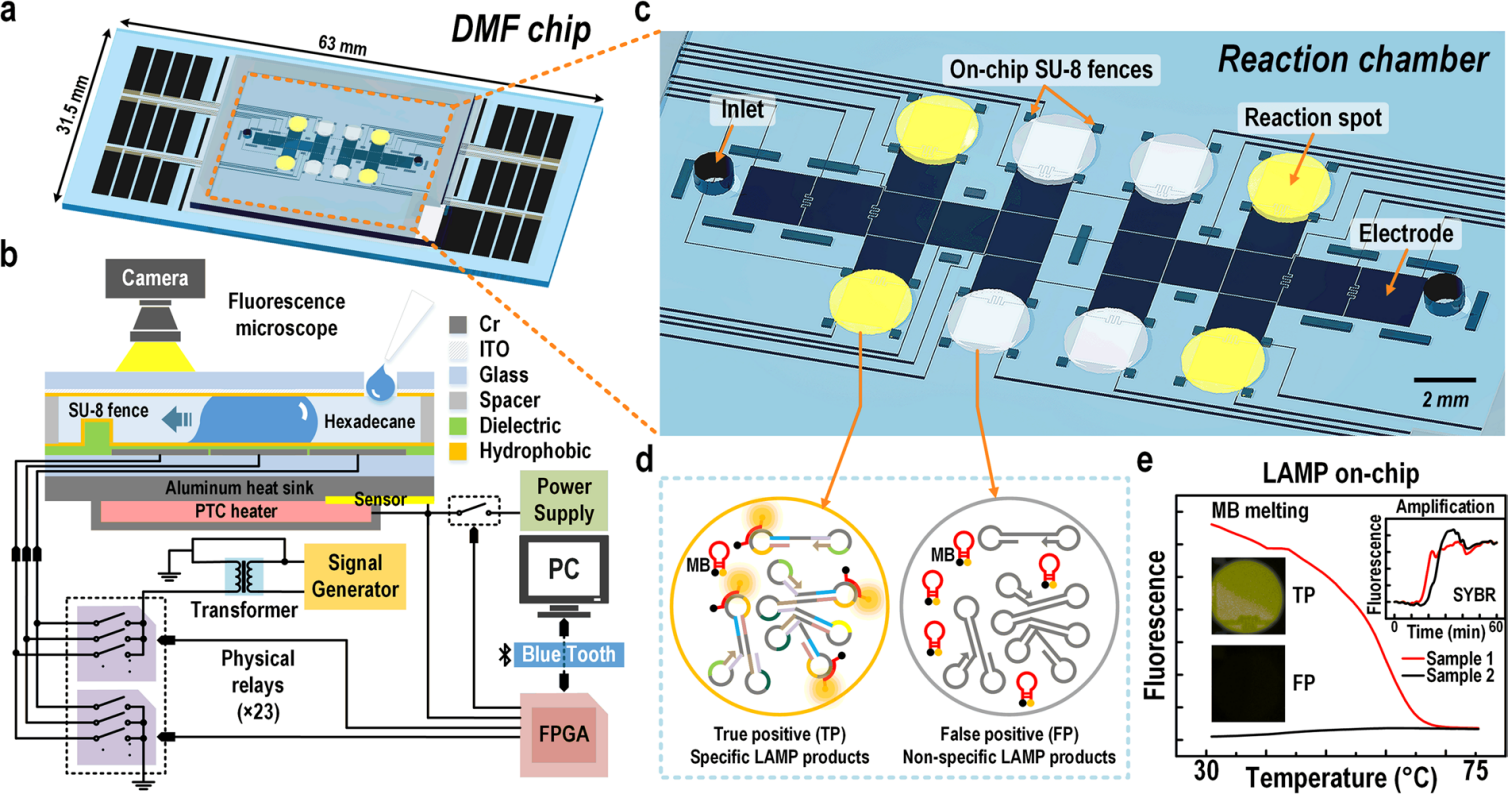
Overview of the digital microfluidic (DMF) system for loop-mediated isothermal amplification (LAMP) reaction and melting curve analysis (MCA) using Molecular Beacon (MB) DNA probes. (**a**) 3D schematic of the DMF chip. (**b**) Side view of the DMF chip and the control electronics of droplet actuation and heating. (**c**) Detailed illustration of the reaction chamber of the DMF chip. (**d**) Schematic of true positive (TP) and false positive (FP) LAMP products with and without MB binding. (**e**) Data example obtained from on-chip LAMP amplification and MCA for TP and FP samples.

In this work, we introduce a DMF LAMP system and its software control^30^ having a sample loading (electric-driven) mode and reaction measuring (heating) mode. In this system LAMP amplification is detected in real-time using SYBR Green I, an intercalating dsDNA dye and product specificity is confirmed at end-point via rapid MCA using a low-T_m_ Molecular Beacon probe (Fig. 1). The low-T_m_ Molecular Beacon probe did not or rarely bound with the target during amplification, and therefore did not hinder the LAMP reaction. In these respects our improvement of LAMP follows the logic of LATE-PCR^27^. DMF LAMP on-chip tests also have the advantage of lowering the risk of aerosol contamination compared to off-chip LAMP assays using a commercial qPCR machine. A LAMP assay targeting repetitive insertion mobile element (RIME) of the human African trypanosomiasis pathogen *Trypanosoma brucei* (*T. brucei*) was exploited as a model assay for pathogen detection^31^. A number of molecular diagnostic methods have been developed to detect *T. brucei*, such as PCR^32,33^, real-time PCR^34^, DNA dipstick test^35^ and conventional LAMP^36^. These methods either require expensive instruments or have great risk in cross-contamination. Regarding to the requirements for pathogen detection in the field, i.e. fast analysis time, low contamination, precise diagnostic results and high sensitivity, our system here has been validated its potential for applications in POCT.

## Results and Discussion

### LAMP assay off-chip optimization

**Figure 2:**
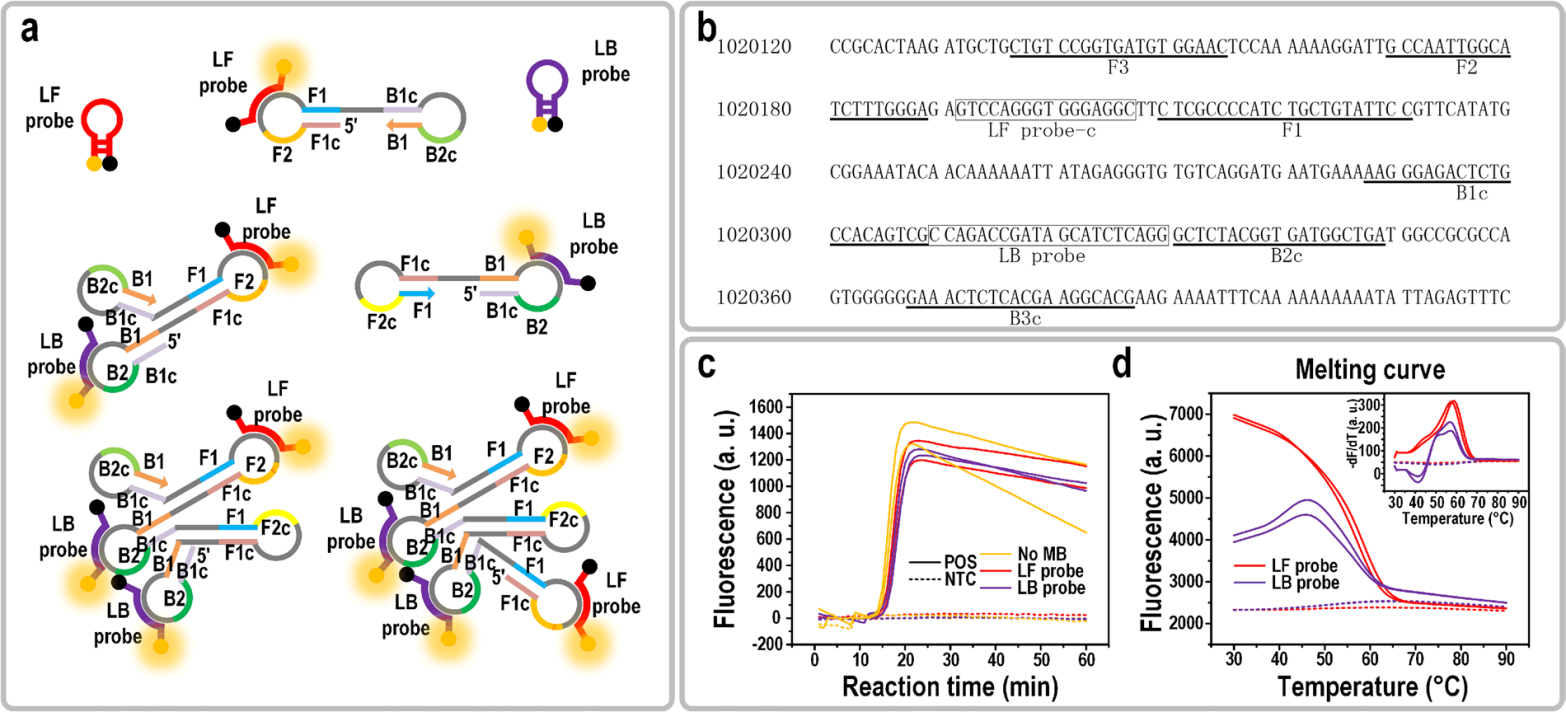
LAMP primers, Molecular Beacon (MB) DNA probes design, and off-chip LAMP reactions. (**a**) Schematic of Molecular Beacon DNA probes and LAMP products (partial) hybridization. (**b**) Part of the nucleic acid sequence of *T. brucei* chromosome 11 and loci of LAMP primers and probes. (**c**) Amplification curves of off-chip optimized LAMP reactions (SYBR Green I fluorescence) with no MB probe, LF probe, or LB probe. LAMP reactions were run in duplicate. (**d**) Off-chip melting curves of LF probe and LB probe after implementing LAMP reactions. Inset shows the melting peaks of LF probe and LB probe.

The LAMP reaction synthesizes DNA via auto-cycling of a DNA polymerase, Bst, having high strand displacement activity^12^. A set of two outer primers (primer F3 and B3) and two inner primers (primer FIP and BIP, consisting of F1c/B1c and F2/B2) are utilized to form dumbbell-structure DNA products as the later template for auto-cycling synthesis (Fig. 2a). Massive LAMP products with multiple single-stranded loop structures are formed as the reaction progresses, enabling the probe hybridization assay to be carried out after the reaction.

In this work, Molecular Beacon probes LF and LB were designed to bind on the strand between F1 and F2, B1 and B2, respectively. Figure 2b shows a partial RIME sequence on chromosome 11 of the genome of *T. brucei*, on which the primers used in this experiment were designed. Other copies of RIME with nucleotide variations in the primer and probe binding sites have also been found in the *T. brucei* genome and have a chance to be detected. The binding locations of the primers and Molecular Beacons, as well as the RIME sequence with nucleotide variation can be found in the Supplementary Fig. S5 and Table S1.

Reaction conditions of the LAMP assay with the Molecular Beacon probes were optimized off-chip. The concentration of both primers and Bst polymerase were double the recommended amount in the datasheet to obtain a faster reaction rate. As shown in Fig. 2c, the amplification could be detected as early as 15 min and plateaued in 5 min for 1,000 copies of DNA per reaction. Similar reaction efficiency was observed for the assays with the LF or LB probe. When comparing the reaction efficiency with the traditional LAMP assay without Molecular Beacon probes, no significant difference was observed, indicating that the Molecular Beacon probes did not inhibit the reaction. We attributed it to the low probe T_m_, which was well below the reaction temperature at 65 °C. The T_m_ of each Molecular Beacon probe was experimentally obtained from the MCA after LAMP reaction, as shown in Fig. 2d. For the LF probe, melting peaks were sharp and distinguishing (inset in Fig. 2d). However, melting peaks for the duplicate samples of the LF probe showed up at 57 °C and 59 °C instead of a unique peak at a constant T_m_. This T_m_ scattering phenomenon was also observed in the subsequent experiments with multiple replicates of one sample or different DNA serial dilutions. The details and possible reasons will be discussed in the following section of “Detection limit of on-chip LAMP”.

As shown in Fig. 2d, the T_m_ of LB probe was 57 °C. There was also a small peak at 51 °C, indicating the presence of other targets for the LB probe with nucleotide variation in the LAMP products. The fluorescence increment (compared to NTC) of the LB probe at the endpoint was distinguishable but was only half as much as that of the LF probe, and the melting curve increased slightly from 30–48 °C. This may have been caused by unexpected secondary structure formation of the LB probe, which needed a high temperature to unwind. Thus, the LB probe was not able to fully bind to its target at 30 °C. The nucleotide variation in the target sequence of LB probe can be found in Supplementary Table S1.

Both LF and LB probe worked nicely by giving distinguishable melting peaks without interfering the LAMP efficiency. Because a single probe would be adequate for all the subsequent experiments, we choose the better one from the two available options. All of the following experiments executed in this paper used the LF probe as the specificity indicator.

### On-chip thermal profiles and DMF chip operation

Two thermal profiles were needed for specific DNA analysis with the Molecular Beacon LAMP assay: a reaction kinetics profile and a melting profile.

**Figure 3:**
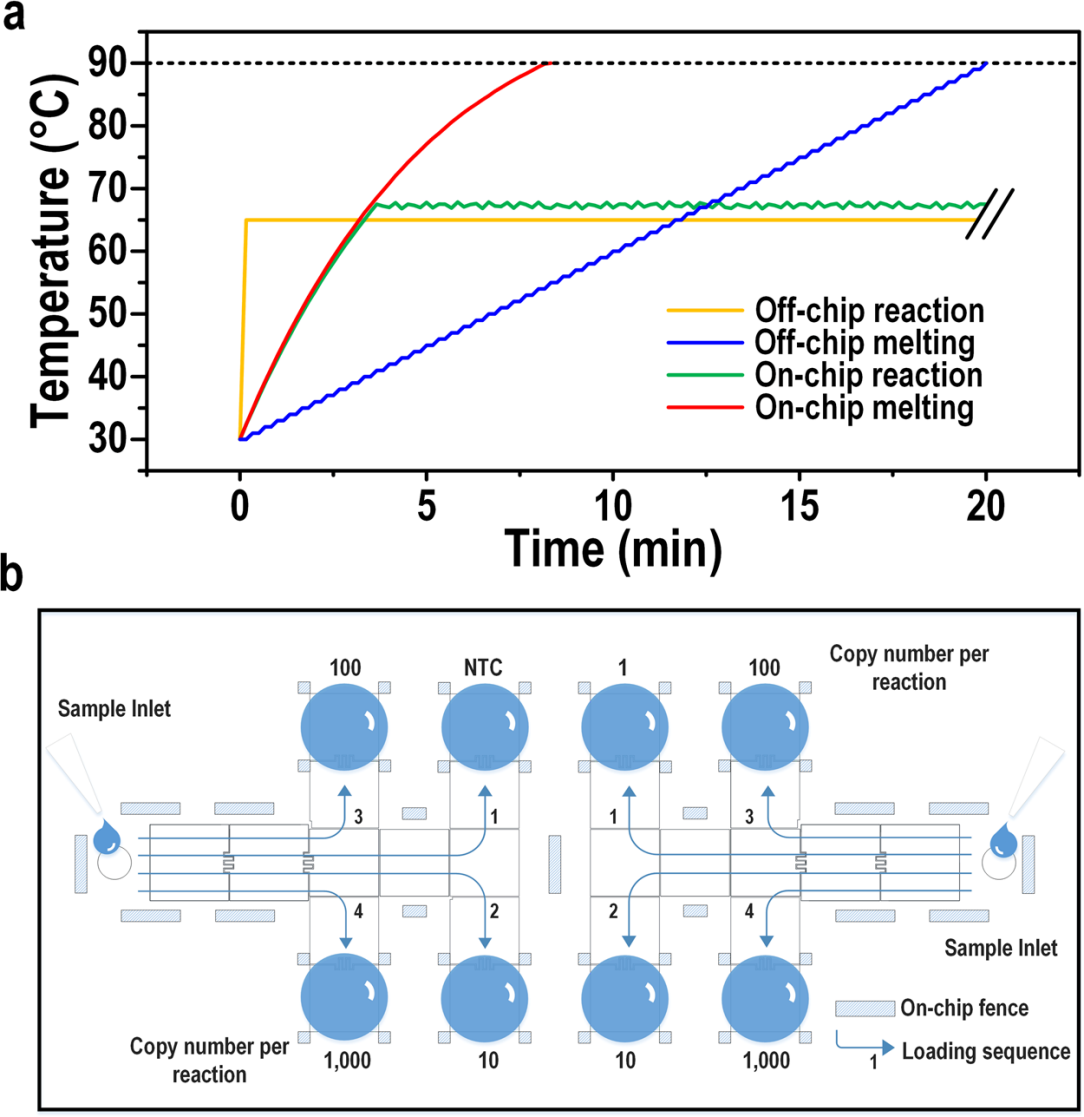
LAMP thermal profiles and on-chip DMF chip operation. (**a**) Reaction and melting profiles of off- and on-chip LAMP. (**b**) Schematic of DMF chip operation for the on-chip LAMP reaction.

Off-chip LAMP reaction occurs at a constant temperature of 65 °C. For the on-chip assay, the reaction droplet (1 μL) was heated by a positive temperature coefficient (PTC) heater in a continuous non-linear manner with a constant DC power input as shown in Fig. 3a. With real-time temperature monitoring through the temperature sensor inserted between the PTC heater and the heat sink, the power was switched off once the temperature increased 0.5 °C over the pre-set value and switched on once the temperature decreased 0.5 °C below the pre-set value. Thus, there was a small fluctuation of ± 0.5 °C stabilizing the on-chip reaction temperature. Considering the heat dissipation, the heater temperature for on-chip LAMP was set slightly higher than 65 °C. Because LAMP reaction tolerates a wide range of temperature (55–68 °C). We tried 67 ± 5 °C and it worked nicely for the on-chip reaction. So we adopted this temperature for the subsequent on-chip experiments.

Off-chip MCA in a qPCR machine adopts a linear step-by-step profile as shown in Fig. 3a. The temperature stabilizes at each interval for a certain time prior to fluorescence capture. Combining the temperature ramping, stabilizing and fluorescence capture time, the off-chip melting takes at least 20 min (for Bio-Rad CFX96^™^) to produce a 30–90 °C melting curve at 1 °C interval. In this case, the temperature resolution was manually set at 1 °C for off-chip MCA. In contrast, on-chip MCA adopts the continuous non-linear heating profile considering the fast heat transfer to a 1-μL droplet. The resolution for on-chip melting depends on the fluorescence capture time by the microscope. In this work, fluorescence was captured every 6 s, which corresponded to an approximate 1 °C change per data capture. The whole on-chip MCA could be accomplished in 8 min, 2.5× shorter than off-chip MCA.

Figure 3b shows the schematic operation of the DMF chip. In order to reduce the loading contamination risk to a minimum, NTC samples and low DNA copy number samples were loaded in first, followed by higher DNA copy number samples.

### On-chip LAMP reaction and MCA

In order to validate the DMF system for on-chip LAMP, four 1-μL replicates from one mother reaction solution with 1,000 copies/1-μL of *T. brucei* genomic DNA templates were run on three different DMF chips and compared with off-chip samples (1,000 copies/10-μL of DNA). The LAMP reaction master mix was prepared off-chip.

**Figure 4:**
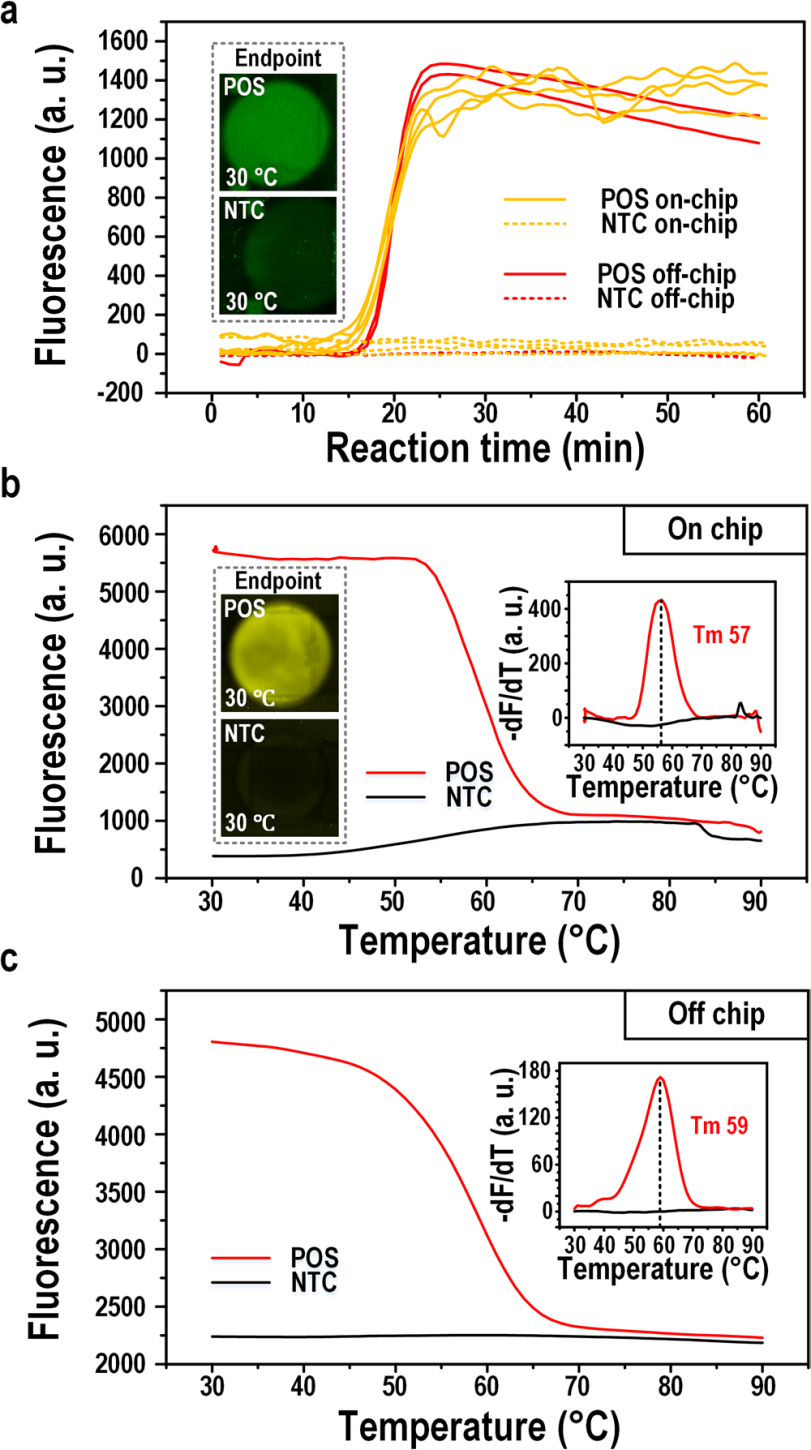
On-chip and off-chip LAMP comparison. (**a**) Amplification curves for on-chip and off-chip LAMP. Inset shows the on-chip endpoint SYBR Green I fluorescence images for a positive and an NTC samples. (**b**) On-chip melting curve for the Molecular Beacon probe. Inset shows the melting peak and the endpoint fluorescence images of Molecular Beacon fluorescence before MCA. (**c**) Off-chip melting curve for the Molecular Beacon probe. Inset shows the melting peak.

Figure 4a shows the real-time fluorescence signals of SYBR Green I. All of the four replicates show very comparable reaction efficiency to their off-chip counterparts (in duplicate), indicating excellent reproducibility across different runs for the on-chip LAMP reaction. The inset in Fig. 4a shows the endpoint SYBR Green I fluorescence after a 1-h on-chip LAMP reaction when the temperature was restored to 30 °C. The positive sample was brighter than the negative control at the endpoint, though with a weak contrast; This was because the SYBR Green I dye had an inhibitory effect on the LAMP reaction^37^, so a low dose of SYBR Green I was added to enable real-time monitoring without inhibition of the reaction. The inhibition effect of SYBR Green I dye was tested and presented in the Supplementary text and Fig. S6. Moreover, a photo-bleaching effect during image capturing throughout the real-time LAMP reaction (120 captures in 1 h) also weakens the endpoint fluorescence of SYBR Green I. Hence, without the real-time curve of the fluorescence change, endpoint SYBR Green I fluorescence was a less convincing measure of LAMP amplification. The photobleaching effect was tested and presented in the Supplementary text and Fig. S7.

It is noticeable that on-chip amplification curve fluctuated after signal plateau, while off-chip amplification curves were smooth. This phenomenon was caused by several factors. In general, the fluorescence signals capture was different for off-chip and on-chip LAMP. Off-chip fluorescence signals were captured and processed by a commercial qPCR machine. On-chip fluorescence signals were captured by a fluorescence microscope and processed manually. First, the light source, resolution of fluorescence capture and the signal data process are different for these two equipments. Second, in an off-chip run, signals of 10-μL reaction solution was more stable than a 1-μL on-chip reaction droplets. Third, a commercial qPCR machine adopts some algorithms to smooth the amplification curve.

In contrast to endpoint SYBR Green I signals, endpoint Molecular Beacon probe signals were much stronger and detectable above background for both off- and on-chip LAMP at 30 °C (inset in Fig. 4b). This may be attributed to the indigenous property that SYBR Green I has a higher fluorescence at 30 °C, the relatively lower concentration of SYBR Green I we added in the sample, and the photobleaching effect of SYBR Green I which underwent more exposures.

After endpoint readouts, MCA was carried out to verify the specificity of the Molecular Beacon probe signals, as shown in Fig. 4b,c. Similar melting curves were produced, and melting peaks culminated at 57 °C and 59 °C for on- and off-chip, respectively, demonstrating the feasibility of on-chip MCA and the specificity of the LAMP reaction.

Furthermore, note that the T_m_ of the Molecular Beacon probe (LF probe) was around 60 °C, and the fluorescence quenched to complete background level at 75 °C; hence, the upper limit of the melting temperature could be reduced to 75 °C in order to save time and power consumption. In this circumstance, the melting analysis time could be further shortened to less than 5 min. These results suggest that the on-chip MCA of LAMP using a Molecular Beacon probe was fast, accurate, and reliable. Movies of the on-chip LAMP reaction and MCA can be found in the Supplementary Movies S2 and S3.

### Detection limit of on-chip LAMP

The detection limit, i.e. the analytical sensitivity, represents the smallest amount of substance in a sample that can accurately be measured by an assay^38^. In this work, a dilution series starting from a certain concentration of purified *T. brucei* genomic DNA were run in real-time LAMP to identify the detection limit as the least concentration that gave a true-positive result. The detection limit of on-chip LAMP on the DMF chip designed with 8 reaction spots (Fig. 3b) was tested and compared with off-chip LAMP.

**Figure 5:**
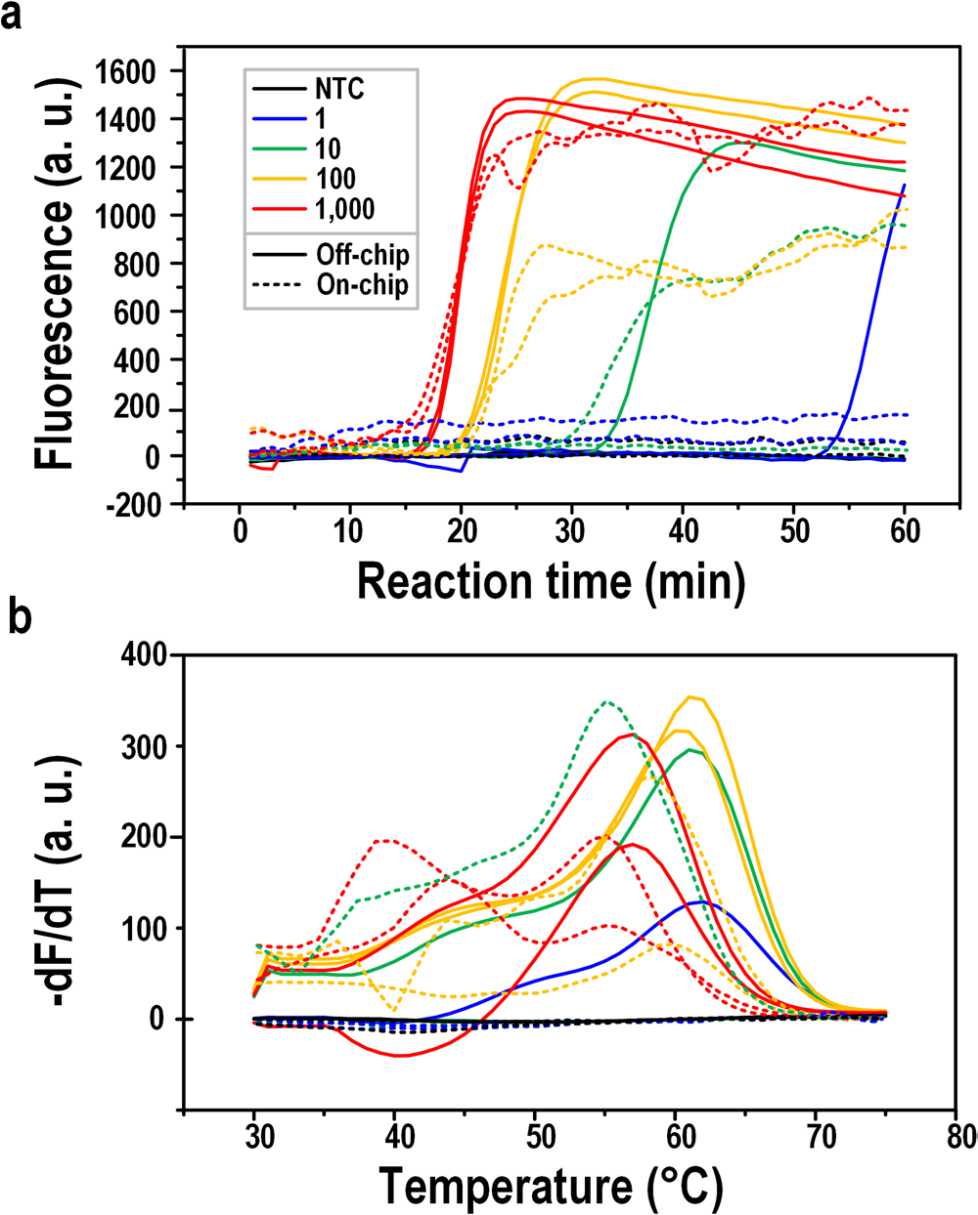
LAMP amplification and melting curves for serial dilutions. (**a**) Off-chip and on-chip LAMP amplification curves of serial dilutions (1–1,000 copies/reaction) for *T. brucei* DNA by SYBR Green I fluorescence. (**b**) Off-chip and on-chip LAMP melting curve analysis by Molecular Beacon probe fluorescence. Serial dilutions were run in duplicate.

As shown in Fig. 5a, off-chip LAMP amplification produced positive signals from 1–1,000 copy of DNA molecules per reaction. The positive rates of off-chip reactions of 10-copy and 1-copy samples were both 1/4. Since the positive off-chip amplification of 10-copy samples was not reproducible, and the 1-copy sample showed a late occurring detectable signal after 50 min, the off-chip amplification of 1-copy was more likely to be sporadic instead of being a solid detection limit. For the on-chip samples, 1-copy of DNA samples on-chip did not produce a positive signal. However, the positive rate of 10-copy samples was 1/2, higher than its off-chip counterpart. We suspected that the on-chip amplification failure of single copy DNA may be due to the limited on-chip reaction spots, which did not offer enough statistical results. Moreover, the lowest detection limit of 10-copy per reaction on-chip plateaued before 40 min. Thus the on-chip LAMP amplification time could be shortened to 40 min, which was adequate to generate a suitably strong fluorescence signal for detection of unknown samples.

Figure 5b shows the MCA of the Molecular Beacon probe for the off-chip and on-chip serial dilutions. Positive melting peaks confirmed that the products of the LAMP amplification were specific, solidifying the comparable detection limitation of off- and on-chip LAMP. As noted before, the melting peaks of the Molecular Beacon probe, i.e., the LF probe, were scattered. Both off-chip and on-chip melting showed a T_m_ range of 56–62 °C. Possible reasons were: (i) different amount of DNA produced in each individual reaction caused difference in the composition of the melting solution, such as concentration of Mg^2+^, dNTP, slightly varying the T_m_ of probe-target melting; (ii) since the target RIME has multiple copies in the genome, there might be nucleotide variations in the probe target; or (iii) the complicated stem-loop structures interfering the probe-target binding. Also, the crowded target products might make the Molecular Beacon probe more difficult to bind or can only partially bind, and therefore decreasing the T_m_. The details of the experiments verifying the possible factors for the scattered melting peaks can be found in Supplementary text, Figs S8 and S9, and Table S2. The concrete reasons are still not crystal clear and will be further studied in more details. However, the T_m_ scattering phenomenon does not affect the Molecular Beacon probe in denoting the specificity of the LAMP product.

Moreover, new melting peaks appeared in on-chip samples with a T_m_ of approximately 40 °C, much lower than the normal T_m_ range. This was not a unique phenomenon for on-chip LAMP, as peaks around 40 °C sometimes appeared in off-chip LAMP as well. This may be due to the multiple RIME regions that can bind to the same probe. In chromosome 11 of *T. brucei gambiense* DAL972, there is another target with 3-nucleotide variation for the LF probe and some nucleotide variations in the flanking primer-binding sequence. Off-chip melting peak around 40 °C and details of the RIME sequences can be found in the Supplementary Fig. S10a and Table S1, respectively.

In order to determine whether the peaks around T_m_ 40 °C were also from specific products, single-stranded DNA targets for the LF probe were designed with a perfect match and a 3-nucleotide mismatch according to the chromosome 11 sequence of the *T. brucei* genome. Results showed that the T_m_ of the LF probe and the perfect match target was 60 °C, while the T_m_ of the LF probe and 3-nucleotide-mismatched target decreased to 41 °C, indicating the off- and on-chip LAMP melting peaks around T_m_ 40 °C came from specific products of the 3-nucleotide mismatched target. The endpoint fluorescence decreased nearly 50% for the 3-nucleotide-mismatched target, but it was still distinguishable from the negative samples. Details of the matched and mismatched probe-target melting analysis can be found in the Supplementary Fig. S10b.

### False positive control

False-positive results were found in both off- and on-chip LAMP with SYBR Green I signals. To verify the capability of our Molecular Beacon probes for ruling out false positive results, end-point fluorescence of both SYBR Green I and the Molecular Beacon probe were compared side by side with on- and off-chip LAMP.

**Figure 6:**
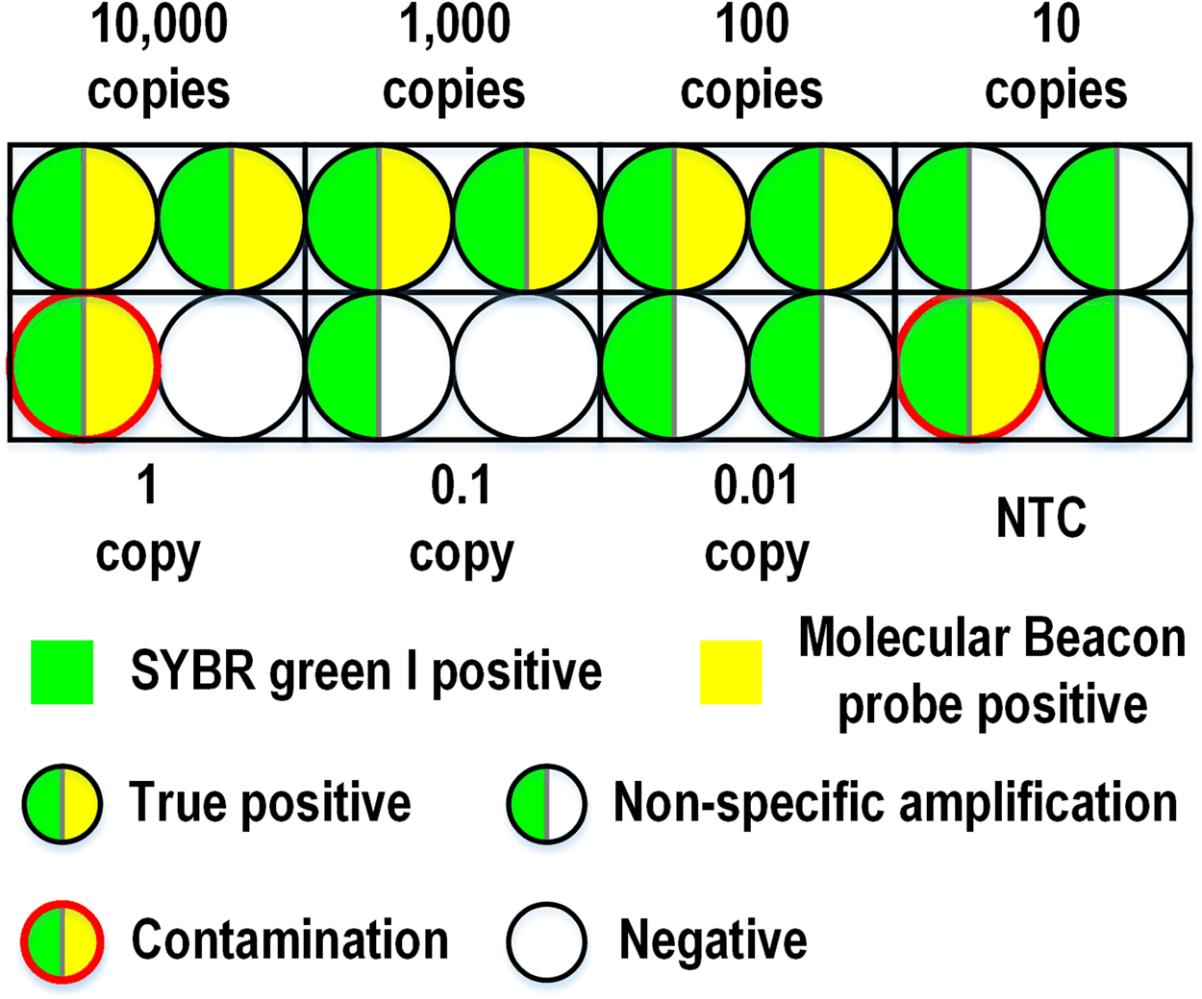
Illustration of off-chip false-positive discrimination. Endpoint amplification results from SYBR Green I signals (green-colored) and specificity results from Molecular Beacon signals (yellow-colored) for the same sample of serial dilutions 0.01–10,000 DNA copies/reaction are shown simultaneously in one circle. Reactions were run in duplicate.

In off-chip circumstances, false-positive results were quite common. As shown in Fig. 6, in one batch of serial dilutions experiments (0.01–10,000 copies/reaction), several samples of low copy numbers, even NTCs, showed positive amplification according to SYBR Green I fluorescence signals. Amplification from NTCs was apparently due to the cross-contamination in this batch of experiments. Six of those products (both of the 10-copy samples and 0.01-copy samples, one of the NTC and 1-copy samples) had late occurring amplification after 40 min, but no probe signals were detected at all. These false-positive results most likely came from the non-specific priming or the primer-dimer formation. However, 2 false-positives, including an NTC, showed probe signals as well but with early amplification SYBR Green I signals (around 30 and 35 min, respectively). These positive results were very likely to be caused by aerosol contamination, which was difficult-to-prevent in an environment where LAMP reactions were performed frequently. LAMP reaction produces a massive amount of DNA, and some DNA molecules can be very long, thus containing countless loci for amplification. As a result, aerosol contamination in LAMP often causes early amplification, in a different manner than PCR where contamination can be distinguished for its late quantification cycle (C_q_). In the off-chip LAMP, hexadecane oil was added into the tube to seal the reaction, and several strict measures were taken to avoid aerosol contamination. However, carryover contamination still frequently occurred. The contamination happened most likely either when dispensing the mixed reaction solution into each reaction tube, as a 10-μL reaction has a large surface area exposed to the air; or inside the qPCR machine, where the hot lid (105 °C) may deform the PCR cap leaving a chance for the aerosol contamination to get into the tube and penetrate the oil layer. The original off-chip amplification curves and melting peaks of false-positive discrimination can be found in the Supplementary Fig. S11.

In contrast, for on-chip LAMP, as little as 1 μL of sample for each reaction was input directly in the reaction chamber filled with hexadecane oil. No air was left in the sealed reaction chamber. The DMF chip setup and loading process greatly reduced the risk of aerosol contamination. In fact, for 24 on-chip LAMP reactions with the loading and reaction on the same bench in the laboratory, no contamination was ever observed in any of the experiments.

**Figure 7:**
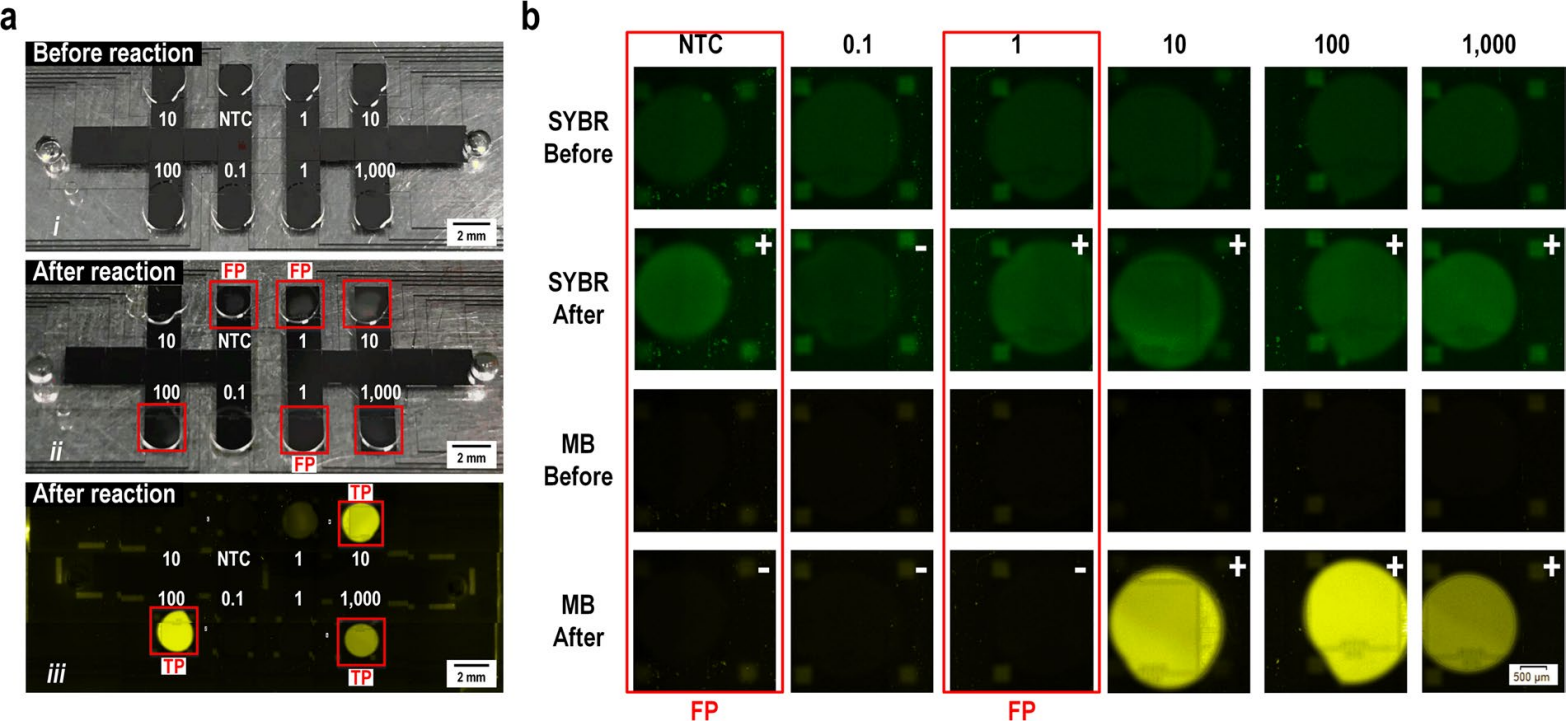
On-chip false-positive discrimination. (**a**) Result comparison of naked-eye LAMP precipitation and Molecular Beacon probe signals. (a-i) Reaction droplets before LAMP reaction under ambient light. (a-ii) Naked-eye visualization of LAMP products after reaction under ambient light. (a-iii) Molecular Beacon probe fluorescence after LAMP reaction under a fluorescence microscope. Red square: alleged positive amplification. FP: false positive. TP: true positive. (**b**) Result comparison of SYBR Green I fluorescence (GFP channel) and Molecular Beacon probe fluorescence (Cy3 channel) before and after LAMP reaction (fluorescence captured under 30 °C). “ + ”: alleged positive. “−”: alleged negative.

On-chip non-specific products, shown in Fig. 7, were also recognized with Molecular Beacon signals. In many clinical uses of LAMP, turbidity is an important and easy way to discern the results of amplification. Under ambient light, precipitation of magnesium pyrophosphate can be observed by the naked-eye for on-chip LAMP (Fig. 7a-ii, marked in the red square), indicating the LAMP reaction took place in these drops. Yet, under Cy3 fluorescence for the Molecular Beacon, only 3 of 6 samples emitted fluorescence (like a real “lamp” being switched on), which were specific products or true-positives (TP in Fig. 7a-iii). The remaining 3 false-positives were possibly products of non-specific priming or primer–dimer formation. When comparing the SYBR Green I fluorescence with the Molecular Beacon probe fluorescence, samples with positive endpoint SYBR Green I signals but negative Molecular Beacon probe signals indicated false-positive results (Fig. 7b).

In summary, the endpoint Molecular Beacon signal is an effective and useful way to distinguish the specific, genuine result of LAMP reactions from false-positive results. Specifically, when combining the Molecular Beacon DNA probes with the DMF LAMP system, aerosol contamination was controlled and false-positive results were not produced.

## Conclusions

A DMF system for false-positive-free LAMP-based pathogen detection using Molecular Beacon DNA probes was developed. This device could run a LAMP reaction with as little as 1 μL of sample with high reproducibility. Both dsDNA dye for real-time amplification monitoring and Molecular Beacon probes for specific product indication can be applied simultaneously in one reaction droplet. This greatly simplified the loading process and avoided post-amplification manipulation, while having double insurance for true positive confirmation. Unknown pathogen detection on-chip can be finished within 40 min with a detection limit of 10 copies/reaction for *T. brucei* genomic DNA. MCA was conducted in less than 5 min (30–75 °C), which was at least 3x faster than the off-chip melting (minimum 15 min). Most importantly, using Molecular Beacon DNA probes to bind to a specific sequence in the products enabled discrimination of non-specific products from true specific products. Superior to dsDNA dye, low-T_m_ Molecular Beacon probes did not interfere with the LAMP reaction, and generated a much stronger fluorescence at the endpoint than that of negative samples. Last, inputting only 1 μL of sample directly into the hexadecane oil-filled closed chamber of a DMF chip greatly reduced the risk of aerosol contamination. In summary, this DMF LAMP system with the detection assay has been revealed as a potential tool for clinical use. The device and all accessories, such as the power supply, signal generator, transformer, and fluorescence microscope can be further miniaturized into a portable size for POCT or field applications.

## Methods

### DMF chip design and fabrication

Arrays of on-chip electrodes (droplet generating electrodes: 2 mm × 2 mm, transport and reaction electrodes: 1.75 mm × 1.75 mm), contact pads, a ground pad and connection wires, photomasks for dielectric layers, a frame spacer and top plate with inlets were designed using AutoCAD (Autodesk). Chromium (Cr) electrodes patterned on glass substrates (63 mm × 63 mm, for two DMF chips) as the bottom plate and chromium (III) oxide (Cr_2_O_3_) glass photomasks were fabricated by Shaoguang Technology (Changsha, China). The PMMA frame spacer with a thickness of 200 μm and the bottom plate segmentation were cut by a laser cutter (ZKJ Tech, China). Inlet holes on the top plate (ITO glass, Kaivo, China) with a diameter of 1.2 mm were drilled by the laser cutting machine as well. 10-μm SU-8 photoresist (MicroChem, USA) was coated on the bottom plate as the dielectric layer, followed by a second layer of patterned SU-8 of 100-μm thickness as on-chip fences to prevent droplet drifting^39^. The details of the on-chip fences fabrication can be found in Supplementary online. A 100-nm Teflon AF (Dupont, USA) layer was coated on both plates to provide hydrophobic surfaces. Finally, the bottom plate, spacer, and top plate were assembled together with liquid metal (Liquid Ultra, Coollaboratory) deposited between the ground pad on the bottom plate and the ITO layer on the top plate. The melting point and boiling point of the liquid metal were 8 °C and 1350 °C respectively. The distance between the liquid metal and the reaction chambers ensured no interference to LAMP reactions. The chip was finally sealed with UV glue. The detailed DMF chip fabrication and assembly illustration can be found in the Supplementary Figs S1 and S2.

### DMF LAMP system setup and operation

The DMF LAMP system incorporates two modes: the loading mode for sample inputting (droplet actuation by electro-wetting force) and the measuring mode for LAMP reaction and MCA (heating).

Both modes share the following three modules: (i) a DMF chip, (ii) an electronic hardware control system—a 3-in-1 device, integrating a DMF chip holder for DMF chip connection to the electronic circuit on a printed circuit board, a PTC ceramic heater with temperature sensor, and a field programmable gate array unit (FPGA) (DE0-nano, Altera, USA) for actuation signal control and data transfer, and (iii) an operation system (in-house customized software) for manual input of droplet actuation and temperature control commands.

The loading mode entailed a high-voltage-generating module in addition to the common modules. The signal generator produced the droplet actuation signal (square wave, 1k Hz), amplified to 70 to 100 V_rms_ by a transformer for droplet transportation.

The measuring mode required a fluorescence measurement module on top of the common modules. In addition, a 12-V DC signal was provided to the PTC heater. A heat sink (Aluminum, 3 mm) was wrapped around the PTC heater to provide an even and flat surface (60 mm × 40 mm × 3 mm) for DMF chip heating. A thin thermocouple sensor was inserted between the heat sink and the heater for real-time temperature sensing.

For system operation, a DMF chip was firstly inserted into the chip holder connected to all of the electronic control modules. Secondly, the loading mode was turned on through the application of a high voltage AC signal. The hexadecane oil medium was infused through the inlets to promote droplet transportation and prevent droplet evaporation during reactions. One microliter of sample was injected through the inlet and pulled into the chamber by the first two energized electrodes. Then, a programmed electrode charging sequence led the droplets to their reaction spots automatically. A movie showing the loading of serial samples is provided in the Supplementary Movie S1. Once all of the samples were loaded, the high-voltage-generating module was disabled for safety reasons. Then, heating and measuring started for the LAMP reactions and MCA with fluorescence capture under the fluorescence microscope. Details of the system setup and operation can be found in the Supplementary Figs S3 and S4.

To minimize the exposure of aerosol contamination in the ambient environment, no outlet for waste collection was designed on-chip, as the presence of an outlet posed greater risk for releasing LAMP products into the air. After the reaction process, the chip was wholly disposed with the inlets tightly sealed by adhesive tape.

### DNA template preparation

*T. brucei* DNA was isolated and purified from mice. Concentration of purified DNA was measured by the Quant-iT™ PicoGreen dsDNA Assay Kit (Invitrogen, USA) and SpectraMax M Series Multi-Mode microplate reader (Molecular Devices, USA) as per the manufacturer’s protocol. DNA serial dilutions were prepared using Tris-EDTA (TE) buffer (Fluka Analytical, Switzerland).

### Off-chip LAMP reaction and MCA

Off-chip and on-chip LAMP reaction solutions were prepared using NEB Bst (New England Biolabs, UK) following an optimized LAMP protocol. For off-chip LAMP, a total volume of 10 μL was prepared and run on a Bio-Rad CFX96™ Real-Time PCR Detection System (Bio-Rad, USA). Primers and Molecular Beacon probes sequences used throughout are listed below:

Forward 3 (F3):

5′-CTGTCCGGTGATGTGGAAC-3′

Backward 3 (B3):

5′-CGTGCCTTCGTGAGAGTTTC-3′

Forward Inner Primer (FIP):

5′-GGAATACAGCAGATGGGGCGAG **GCCAATTGGCATCTTTGGGA**-3′

Backward Inner Primer (BIP):

5′-AAGGGAGACTCTGCCACAGTCG **TCAGCCATCACCGTAGAGC**-3′

(Underlined: F1c/B1c sequence; **Bold and underlined**: F2/B2 sequence. *T. brucei* RIME primers F3/B3 and FIP/BIP come from Njiru *et al*.^31^).

Molecular Beacon looped probe F (LF probe):

5′-Cy3-ATGCCTCCCACCCTGGACAT-BHQ2-3′

Molecular Beacon looped probe B (LB probe):

5′-Cy3-ATCCAGACCGATAGCATCTCAGGAT-BHQ2-3′

(Underlined: Targeted probe sequence. Flanked: non-target for stem formation on both ends).

All the primers and probes were purchased from Genewiz (Suzhou, China).

The final optimized reaction mix contained 20 mM Tris-HCl, 10 mM (NH_4_)_2_SO_4_, 10 mM KCl, 8 mM MgSO_4_, 0.1% TritonX-100 [from stock of 10 × NEB ThermoPol Buffer], 1.4 mM of each dNTP (Applied Biosystems, UK), 0.4 × SYBR Green I [from 10 × stock] (Life Technologies, USA), 400 nM of the Molecular Beacon LF probe or LB probe, 0.64 U/μL of Bst. The primer set contained 0.4 μM of each outer primer, F3 and B3, and 1.6 μM of each inner primer, FIP and BIP. The final reaction volume containing 0.4 μL of DNA template or 0.4 μL of TE for no-template-control (NTC) was adjusted to 10 μL with ddH_2_O and sealed with a drop of hexadecane (Sigma-Aldrich, Germany) and the PCR tube cap. Each reaction was run in duplicate at 65 °C for 60 min (fluorescence recorded every 1 min), followed by a step at 85 °C for 5 min to inactivate the Bst. MCA was conducted after the stabilization at 30 °C for 5 min, followed by melting from 30 °C to 90 °C with a 1 °C interval for 2 s (fluorescence capture time was approximately 12 s per step).

### On-chip LAMP reaction and MCA

The reaction mix for on-chip LAMP was identical to that for off-chip LAMP. One tenth of the volume (1 μL) of the off-chip LAMP reaction (10 μL) was loaded onto the chip and driven to the reaction spots. Reactions were run at 67 ± 0.5 °C for 60 min. A fluorescence microscope (Olympus DP80, Japan) was used to monitor the SYBR Green I signals in real-time and record the fluorescence signals every 30 s with an exposure time of 0.5 s (GFP channel). After reaction, the temperature was restored to 30 °C and maintained for at least 5 min before end-point probe signal readouts (Cy3 channel, exposure time 0.8 s). Because hexadecane oil expands upon high temperature which may cause droplets drifting, so no deactivation at 85 °C for 5 min was adopted for on-chip reactions. Instead, the 30–90 °C MCA was capable of deactivate the Bst for 1-μL on-chip droplets. For performing the MCA, the temperature was raised from 30 °C to 90 °C continuously. Temperature was recorded every 0.1 s with the temperature sensor. Fluorescence signals were captured every 6 s (Cy3 channel, exposure time 0.8 s) and synchronized with the temperature reading. All of the fluorescence signals were captured in a dark environment.

### Data availability statement

The datasets generated during and/or analyzed during the current study are available from the corresponding author on reasonable request.

## Electronic supplementary material

Supplementary Information

Sample loading on digital microfluidic chip

Loop-mediated isothermal Amplification on digital microfluidic chip

Melting curve analysis by molecular beacon probe on digital microfluidic chip
