# Supplementary material for: A digital microfluidic system for loop-mediated isothermal amplification and sequence specific pathogen detection
Source: Sci Rep. 2017 Nov 6;7:14586. doi: 10.1038/s41598-017-14698-x (PMC5673945; doi:10.1038/s41598-017-14698-x)
Supplement: Supplementary file 1 — Supplementary Information [file 41598_2017_14698_MOESM1_ESM.pdf]

Supplementary Information

A digital microfluidic system for loop-mediated isothermal amplification and sequence specific pathogen detection.

Liang Wan[1,2], Tianlan Chen[1], Jie Gao[1], Cheng Dong[1,2], Ada Hang-Heng Wong[3], Yanwei Jia[1,*], Pui-In Mak[1,2], Chu-Xia Deng[3] and Rui P. Martins[1,2,4]

[1] State-Key Laboratory of Analog and Mixed-Signal VLSI, University of Macau, Macao, China.
[2] Department of ECE, Faculty of Science and Technology, University of Macau, Macao, China.
[3] Cancer Centre, Faculty of Health Sciences, University of Macau, Macao, China.
[4] On Leave from Instituto Superior Técnico, Universidade de Lisboa, Portugal.
* This indicates correspondence author(s).

Correspondence should be addressed to:
Name: Yanwei Jia, PhD
Phone: +853 - 8822 4451
Email: yanweijia@umac.mo

1. DMF chip fabrication and Assembly

On-chip fences fabrication

Including the LAMP reaction and MCA, the entire DNA detection process lasted about 1 h, during which droplets might drift in an unpredictable manner from their reaction spots owing to the highly hydrophobic surface of the top and bottom plates. To affix the droplet at a specific position, different methods can be applied including continuously charging specific electrodes or changing the hydrophobicity of the surface on certain electrodes[1, 2]. However, these measures can cause electrode breakdown or can reduce reaction efficiency if DNA and protein molecules adhere to the hydrophilic surface and lose amplification activity. In this work, we addressed the issue in a more physical way. On one hand, a spacer of 200 μm height was utilized to increase the surface/height ratio of on-chip droplets, which increased the friction against the top and bottom plate. On the other hand, on-chip SU-8 fences of an approximate 100-μm height were designed and fabricated around the transport and reaction electrodes. These implements successfully prevented the droplets from drifting during sample loading and constrained the droplets at their reaction spots during reactions.

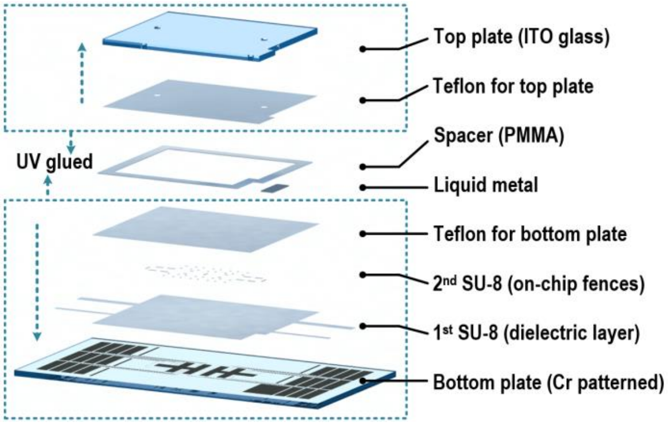

Supplementary Figure S1. 3D schematic of the DMF chip fabrication. The top plate with ITO downward was spin-coated with a thin layer of Teflon (~100 nm). The Cr-patterned bottom plate (wet etched) was spin-coated with 2 layers of SU-8, and 1 thin layer of Teflon. Upon sealing of the whole chip, liquid metal was deposited on the Cr ground pad on the bottom plate (outside the sealed chamber) for connecting the ground pad (bottom plate) with the ITO layer (top plate). Finally, the top plate, PMMA frame spacer (200 μm) and bottom plate were aligned and UV glued.

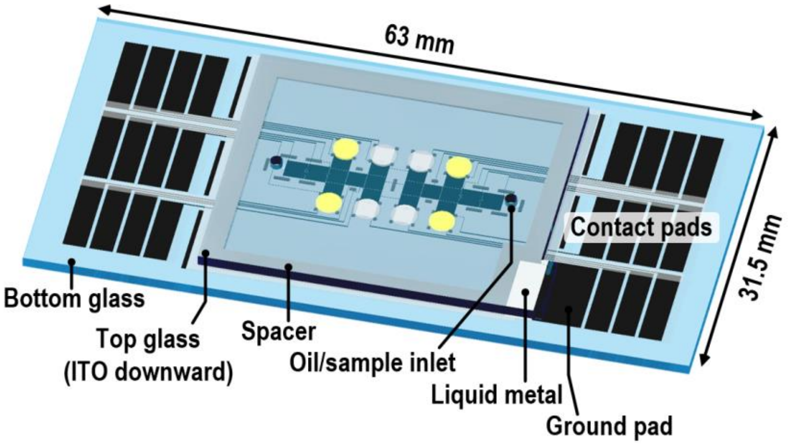

Supplementary Figure S2. Details illustration of the assembled DMF chip.

2. System setup

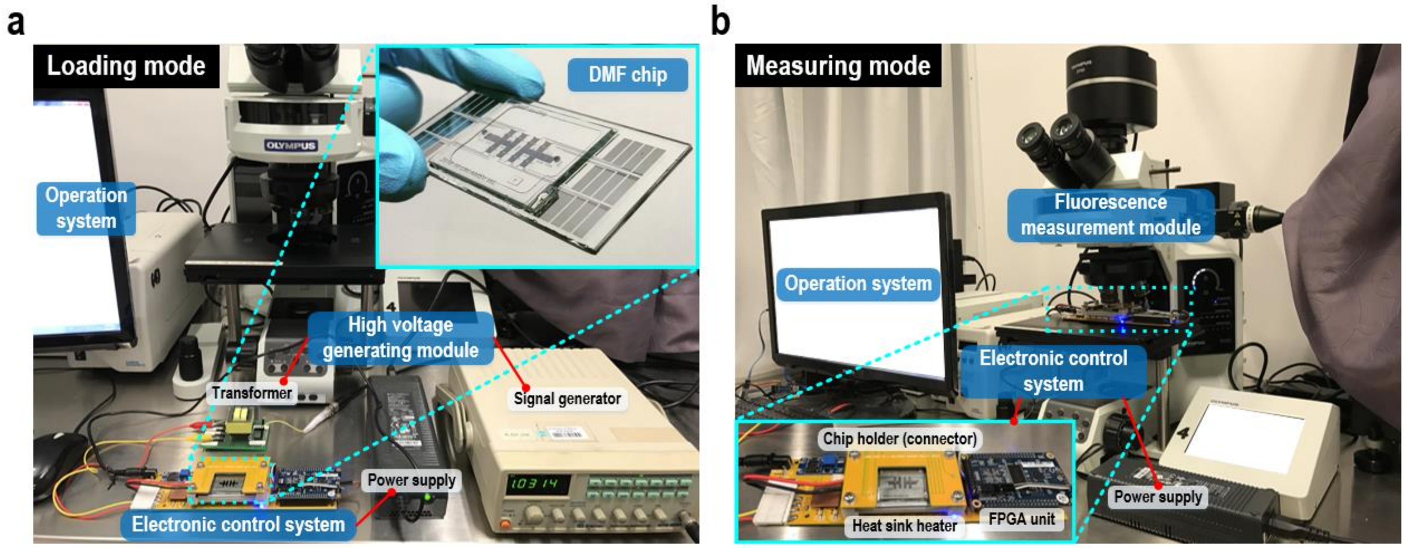

Supplementary Figure S3. Operation and experiment setup of the DMF LAMP system. (a) The loading mode (for sample loading) of the system comprises a electronic control system (works as the electrical connection and control electronics for task execution and signal acquisition), a signal generator and a transformer for high voltage generation, plus a operation system (in-house computer software) for actuation control; (b) The measuring mode (for LAMP reaction and MCA) consists of a electronic control system (works as a heater with a temperature sensor for temperature control), a fluorescence microscope for images capture, and an operation system for temperature regulation, data acquisition and capture of fluorescence images.

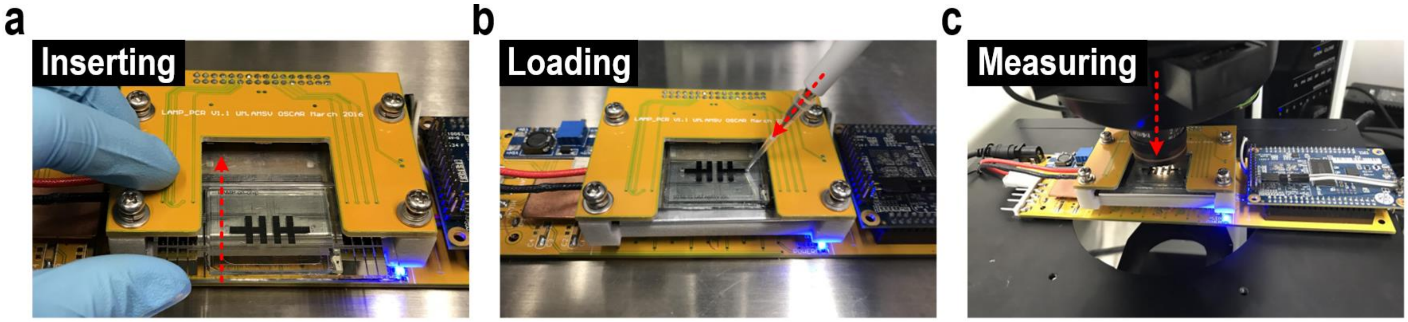

Supplementary Figure S4. Steps of DMF LAMP system operation. (a) Inserting DMF chip into the chip holder for electric control connection. (b) Loading the medium oil and samples under the loading mode. (c) Microscope focusing and LAMP reaction start-up under the measurement mode.

3. LAMP assay

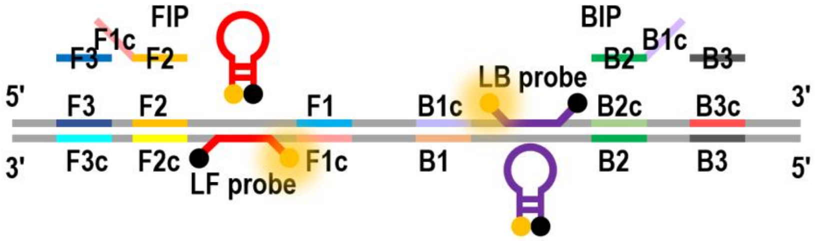

Supplementary Figure S5. Schematic of the LAMP template sequence, primers and Molecular Beacon DNA probes.

Inhibition effect of SYBR Green I

The off-chip reaction mix contained 20 mM Tris-HCl, 10 mM $(NH_4)_2SO_4$, 10 mM KCl, 8 mM $MgSO_4$, 0.1% TritonX-100 [from stock of 10× NEB ThermoPol Buffer], 1.4 mM of each dNTP (Applied Biosystems, UK), 0.4×, 0.8×, 2× and 4× SYBR Green I [from 10×, 20×, 50× and 100× stock] (Life Technologies, USA), 400 nM of the Molecular Beacon LF probe or LB probe, 0.64 U/μL of Bst. The primer set contained 0.4 μM of each outer primer, F3 and B3, and 1.6 μM of each inner primer, FIP and BIP. The final reaction volume containing 0.4 μL of DNA template (10,000 copies/reaction) or 0.4 μL of TE for no-template-control (NTC) was adjusted to 10 μL with ddH_2O and sealed with a drop of hexadecane (Sigma-Aldrich, Germany) and the PCR tube cap. Each reaction was run in duplicate at 65 °C for 60 min (fluorescence recorded every 1 min), followed by a step at 85 °C for 5 min to inactivate the Bst. MCA was conducted after the stabilization at 30 °C for 5 min, followed by melting from 30 °C to 90 °C with a 1 °C interval for 2 s (fluorescence capture time was approximately 12 s per step).

To demonstrate the inhibition effect of SYBR Green I dye, off-chip LAMP reactions with 2-fold (0.8×), 5-fold (2×), and 10-fold (4×) of the concentration of SYBR Green I dye we used in the manuscript were run. As Figure S6a shows, 2-fold (0.8×) of SYBR Green I caused a delay of the reaction by 5.6 min comparing to 0.4×. Melting peaks in Figure S6b confirmed the products to be true positive. 5-fold (2×) and above of SYBR Green I completely inhibited the LAMP reaction. The NTCs of both samples of 0.4× and 1 NTC of 0.8× showed positive amplification curve but negative Molecular Beacon probe signals (Figure S6b), indicating non-specific amplification.

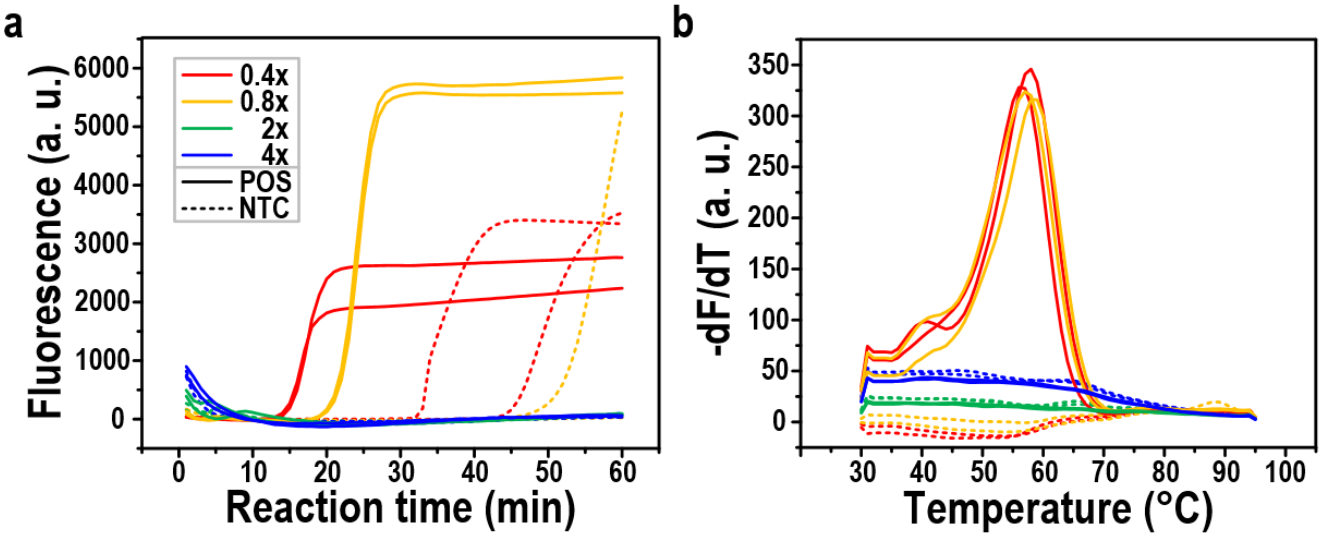

Supplementary Figure S6. Inhibition effect of SYBR Green I dye. (a) Off-chip amplification curves of SYBR Green I signals. (b) Melting peaks of Molecular Beacon probe signals. Reactions were run in duplicate.

Photobleaching effect of SYBR Green I

The reaction mix for on-chip LAMP was identical to that for off-chip LAMP. One 1-μL reaction droplet was loaded on the DMF chip for continuous photobleaching test. Four 1-μL reaction droplets were loaded onto the chip and driven to the reaction spots for LAMP reactions. Two were positive samples containing 1,000 copies of DNA per reaction droplet. Two were NTCs. A fluorescence microscope (Olympus DP80, Japan) was used to monitor the SYBR Green I signals. For continuous photobleaching test, the droplet was exposed under green light from GFP channel for 120 s (signal recorded continuously). For on-chip LAMP reactions, fluorescence signals were captured every 30 s with an exposure time of 0.5 s (GFP channel). Started at 30 °C, the temperature rised up to 67 °C. After reaction for 60 min, temperature was restored to 30 °C. The whole capture time was 85 min. All of the fluorescence signals were captured in a dark environment.

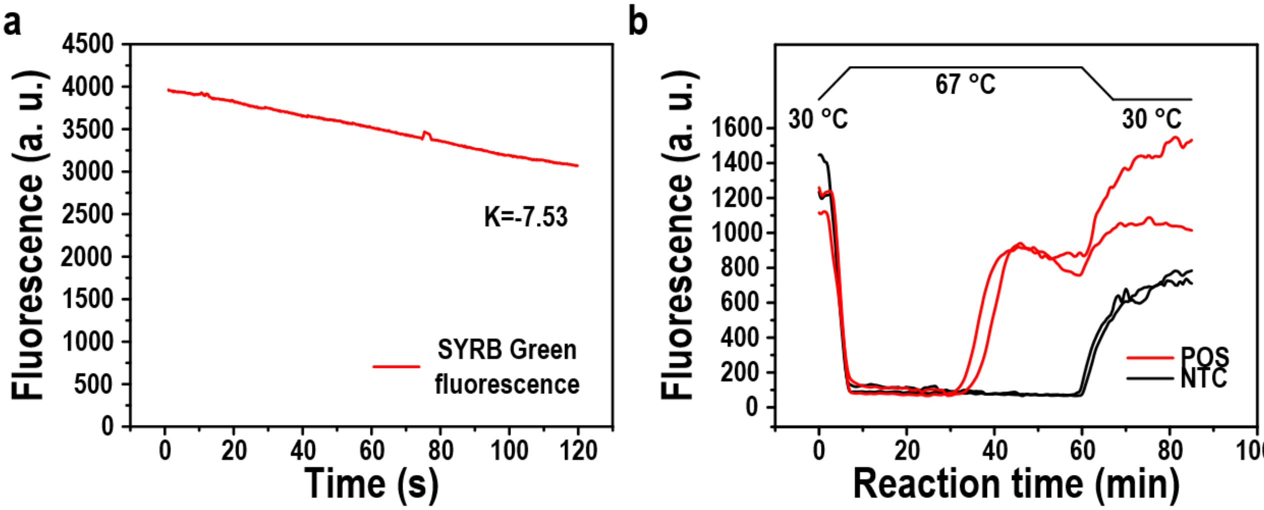

Supplementary Figure S7. Photobleaching effect of SYBR Green I dye. (a) Continuous photobleaching effect. (b) SYBR Green I amplification curves of on-chip LAMP reactions.

Possible reasons of LF probe T_m scattering phenomenon

(i) Complicated composition of the melting solution

The synthetic target sequences of *T. brucei* RIME for Molecular Beacon LF probe are listed below:

Perfect match target (PM):

5'-GGGAGAGTCCAGGGTGGGAGGCTTCTCG-3'

(Underlined: LF probe complementary sequence.)

DNA oligo was purchased from Genewiz (Suzhou, China).

The final melting mix contained 20 mM Tris-HCl, 10 mM $(NH_4)_2SO_4$, 10 mM KCl, 2 mM $MgSO_4$, 0.1% TritonX-100 [stock 10× NEB ThermoPol Buffer], 0 or 6 mM of $MgSO_4$ (total Mg^{2+} concentration was 2 mM or 8 mM). 0 mM or 1.4 mM each dNTP and 400 nM Molecular Beacon LF probe. Final melting volume contained 1 μM DNA target (PM), or equivalent volume of TE for no template control (NTC), adjusted to 10 μL with ddH_2O and sealed with a drop of Hexadecane as well as the PCR tube cap. Melting curve analysis was run with the profile of 95 °C 2 min, 30 °C 5 min, 30–90 °C melting with 1 °C interval for 2 s (fluorescence recorded).

Different concentration combination of Mg^{2+} and dNTP were tested. We analyzed the melting curve of LF probe and the synthesized single-stranded DNA target binding. As Table S2 and Figure S8 show, T_m raises along with the Mg^{2+} concentration increases, whereas T_m goes down when the dNTP concentration increases. The concentrations of Mg^{2+} (8 mM), dNTP (1.4 mM) and the product mass in the LAMP reaction are in a high level, so the slight shifting in T_m is reasonable.

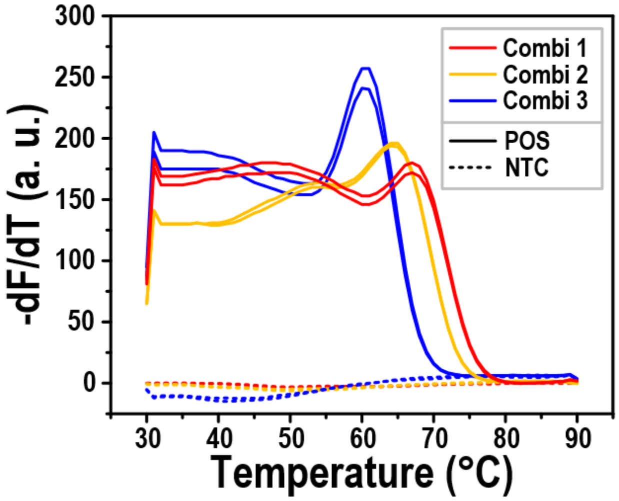

Supplementary Figure S8. Melting curve analysis of LF probe and synthesized DNA target. Combination 1 (Combi 1): Mg^{2+} 8 mM, dNTP 0 mM. Combination 2 (Combi 2): Mg^{2+} 2 mM, dNTP 0 mM. Combination 3 (Combi 3): Mg^{2+} 2 mM, dNTP 1.4 mM. POS: positive (synthesized DNA target 1 μM). NTC: no template control (TE buffer).

(ii) Nucleotide variations in LF probe target

To verify the T_m scattering is caused by the probe-target binding instead of the Molecular Beacon probe-LAMP assay, we used LB probe to do the serial dilutions under the same reaction condition as the LF probe. The target of LB probe has less nucleotide variations than the target of LF probe (Table S1). As Figure S9 shows, the melting peaks are much more centralized (57–58 °C). This result strongly supports that the T_m scattering phenomenon of LF probe is caused by the target nucleotide variations.

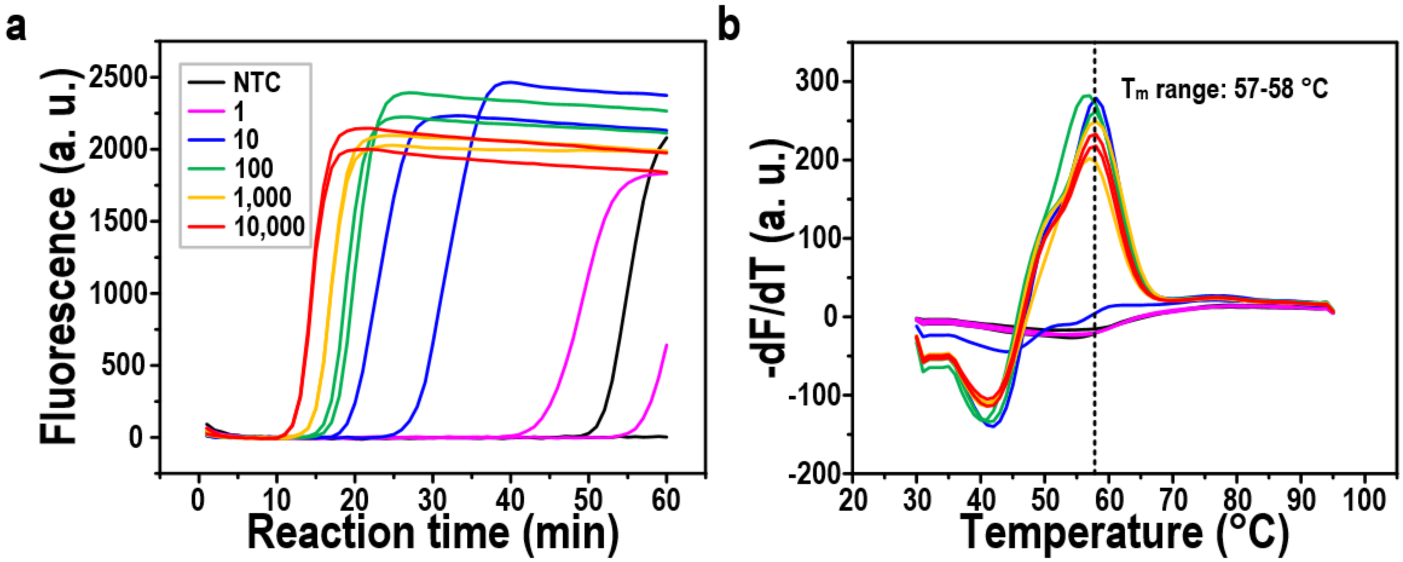

Supplementary Figure S9. Off-chip LAMP amplification and melting curves for serial dilutions for LB probe. (a) LAMP amplification curves of serial dilutions (1–10,000 copies/reaction) for *T. brucei* DNA by SYBR Green I fluorescence. (b) LAMP melting curve analysis by LB probe fluorescence. Serial dilutions were run in duplicate.

In conclusion, the T_m scattering phenomenon of LF probe is mainly caused by the nucleotide variations in the target sequence, which is a very unique case of this model system. This can be prevented by staying away from the unconserved sequence in the primer and probe design step. Also, the complexity of the melting solution contributes in the T_m scattering, causing a tolerable small range shifting of the T_m (1–2 °C). These do not affect the MB probe in denoting the specificity of the LAMP product.

Molecular Beacon probe-target melting curve analysis for alternative target

The synthetic target sequences of *T. brucei* RIME for Molecular Beacon LF probe are listed below:

Perfect match target (PM):

5'-GGGAGAGTCCAGGGTGGGAGGCTTCTCG-3'

3-nucleotide mismatch target (3-mis):

5'-TGGAGAGTCCGGAGTGGGGGGCTTCTCG-3'

(Underlined: LF probe complementary sequence. Double-underlined: nucleotides mismatched with LF probe)

All DNA oligos were purchased from Genewiz (Suzhou, China).

The final melting mix contained 20 mM Tris-HCl, 10 mM $(NH_4)_2SO_4$, 10 mM KCl, 2 mM $MgSO_4$, 0.1% TritonX-100 [stock 10× NEB ThermoPol Buffer], 1.4 mM each dNTP and 400 nM Molecular Beacon LF probe. Final melting volume contained 1 μM DNA target (PM or 3-mis), or equivalent volume of TE for no template control (NTC), adjusted to 10 μl with ddH_2O and sealed with a drop of Hexadecane as well as the PCR tube cap. Melting curve analysis was run with the profile of 95 °C 2 min, 30 °C 5 min, 30–90 °C melting with 1 °C interval for 2 s (fluorescence recorded).

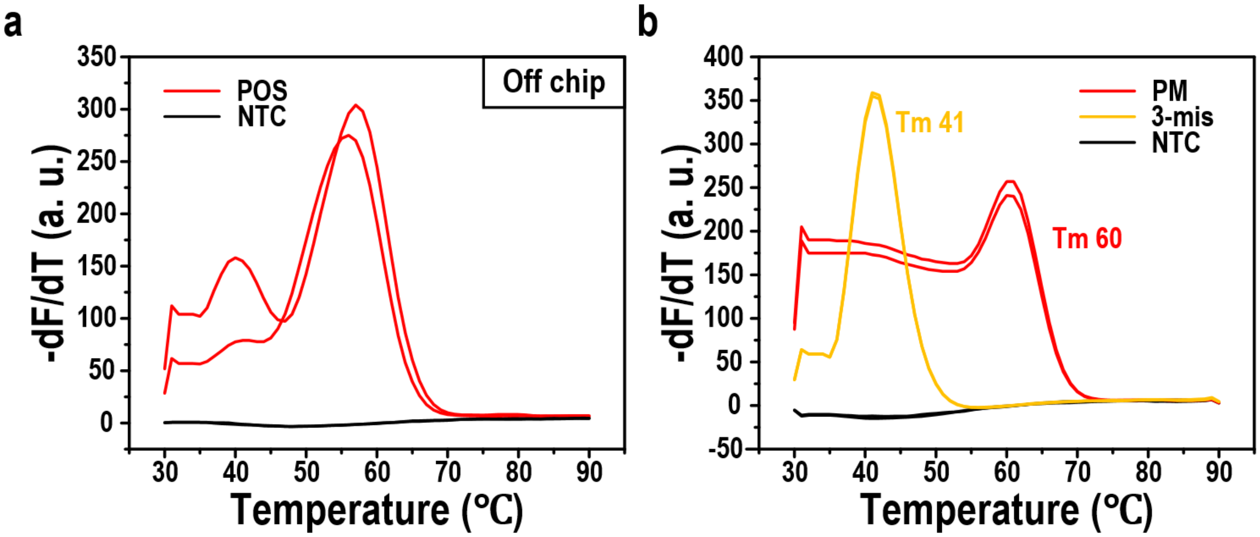

Supplementary Figure S10. (a) Melting peaks of off-chip LAMP products for 10,000 copies/reaction. Reactions were run in duplicate. (b) Melting peaks (off-chip) of Molecular Beacon LF probe and its perfect matched target (PM) and 3-nucleotide mismatched target (3-mis). Reactions were run in duplicate.

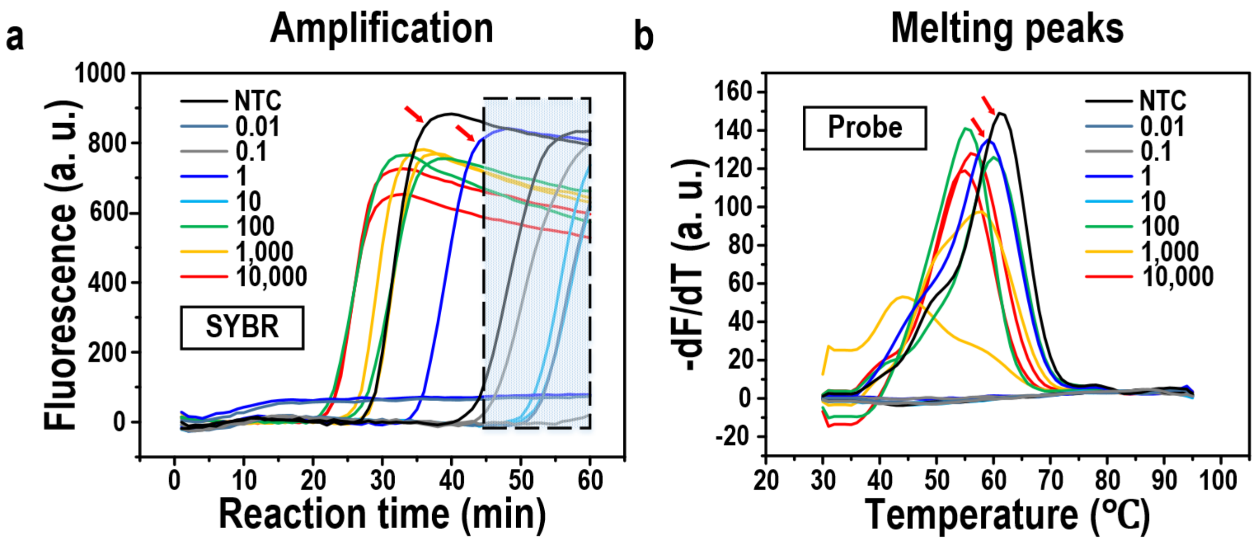

Supplementary Figure S11. Amplification and melting curve analysis of serial dilutions off-chip. Reactions were run in duplicate. (a) Amplification curves of SYBR Green I fluorescence. Dotted line square: non-specific amplification. Red arrows: amplifications by contamination. (b) Melting peaks of Molecular Beacon probe fluorescence. Red arrows: amplifications by contamination.

Supplementary Table S1. Sequence of RIME target in *Trypanosoma brucei gambiense* DAL972 chromosome 11.

Loci	***Sequence (5' – 3')***
150,161-150,411	CTGTCCGGTGATGTGGAACACAAAAAAAGGATTGC**T**AATTG**A**CATCTTT**T**GGAGAGTCC**G**G**A**GTG<br>GG**G**GGCTTCTCGCCCCATCTGCTGTATTCCGTTCAACTGCGGAGCTACAACAAAAATTATAGAGG<br>GAGTGTTAGGATGAATAAAAAAGGGAGACTCTGCCACAGTCGCCAGACCGATAGCATCTCAGGGC<br>TCTACGGTGATGGCTGATGGCCGCGCCAGTGGGGGGAAACTCTCACGAAGGCACG
1,020,135-1,020,386	CTGTCCGGTGATGTGGAACTCCAAAAAAAGGATTGC**T**AATTG**A**CATCTTTGGGAGAGTCCAGGGTG<br>GGAGGCTTCTCGC**T**CCATCTGCTGTATTCCGTTCATATGCGGAAATACAACAAAAAATTATAGAGGG<br>TGTGTCAGGATGAATGAAAAAGGGAGACTCTGCCACAGTCGCCAGACCGATAGCAT**T**TCAGGGCT<br>CTACGGTGATGGCTGATGGCCGCGCCAGTGGGGGGAAACTCTCA**T**GAAGGCACG
1,089,336-1,089,586	CTGTCCGGTGATGTGGAACTCCAAAAAAAGGATTGC**T**AATTG**A**CATCTTT**T**GGAGAGTCC**G**G**A**GTG<br>GG**G**GGCTTCT**T**GCCCCATCTGCTGTTTCCGTTCATATGCGGAAATACAACAAAAATTATAGAGGGT<br>GTGTATTAGGATGAATGAAAAAGGGAGACTCTGCCACAGTCGCCAGACCATAGCATCTCAGGGCT<br>CTACGGTGATGGCTGATGGCCGCGCCAGTGGGGGGAAACTC**C**CACGAAGGCACG
1,105,054-1,105,306	CTGTCCGGTGATGTGGA**C**CTCCAAAAAAAGGATTGCCAATTGGCATCTTTGGGAGAGTCCAGGGTG<br>GGAGGCTTCTCGCCCCATCTGCTGTATTCCGTTCATATGCGGAAATACAACAAAAATTATAGAGGGT<br>GTGTATTAGGATGAATGAAAAAGGGAGACTCTGCCACAGTCGCCAGACCGATAGCATCTCAGGGC<br>TCTACGGTGATGGCTGATGGCCGCGCCAGTGGGGGGAAACTC**C**CACGAAGGCACG

Note: Underlined: sequences of primers for LAMP. Highlighted (blue): LF probe target sequence. Highlighted (green): LB probe target sequence. **Red bold**: nucleotide variation from LAMP primers and probes.

Supplementary Table S2. Melting curve analysis data of LF probe and synthesized DNA target.

	Mg^{2+} conc. (mM)	dNTP conc. (mM)	T_m (°C)
**Combination 1**	8	0	67
**Combination 2**	2	0	64
**Combination 3**	2	1.4	60

Supplementary Movie S1. Sample loading on digital microfluidic chip

One microliter of sample was injected through the inlet and pulled into the chamber by the first two energized electrodes. Then, a programmed electrode charging sequence led the droplets to their reaction spots automatically.Video was accelerated by 5 folds.

Supplemementary Movie S2. Loop-mediated isothermal Amplification on digital microfluidic chip

Time-lapse images and amplification curves of real-time on-chip LAMP reactions. SYBR Green I fluorescence signals was recorded every 30 s with an exposure time of 0.5 s (GFP channel).

Supplementary Movie S3. Melting curve analysis by molecular beacon probe on digital microfluidic chip

Time-lapse images and melting curves of on-chip melting curve analysis of LAMP products. Molecular Beacon probe fluorescence was recorded every 6 s with an exposure time of 0.8 s (Cy3 channel).

References

1. Eydelnant, I.A., Uddayasankar, U., Li, B., Liao, M.W. & Wheeler, A.R. Virtual microwells for digital microfluidic reagent dispensing and cell culture. *Lab Chip.* **12**, 750-757 (2012).
2. Grissom, D.T., McDaniel, J. & Brisk, P. A Low-Cost Field-Programmable Pin-Constrained Digital Microfluidic Biochip. *IEEE T Comput Aid D.* **33**, 1657-1670 (2014).
